# Artificial intelligence–based models for the qualitative and quantitative prediction of a phytochemical compound using HPLC method

**DOI:** 10.3906/kim-2003-6

**Published:** 2020-10-26

**Authors:** Abdullahi Garba USMAN, Selin IŞIK, Sani Isah ABBA, Filiz MERİÇLİ

**Affiliations:** 1 Department of Analytical Chemistry, Faculty of Pharmacy, Near East University Nicosia Turkish Republic of Northern Cyprus; 2 Department of Physical Planning Development, Maitama Sule University, Kano Nigeria; 3 Department of Phytotherapy, Faculty of Pharmacy, Near East University, Nicosia Turkish Republic of Northern Cyprus

**Keywords:** High performance liquid chromatography, retention time, isoquercitrin, artificial intelligence, multilinear regression

## Abstract

Isoquercitrin is a flavonoid chemical compound that can be extracted from different plant species such as *Mangifera indica* (mango), *Rheum nobile* , *Annona squamosal* , *Camellia sinensis* (tea), and coriander ( *Coriandrum sativum* L.). It possesses various biological activities such as the prevention of thromboembolism and has anticancer, antiinflammatory, and antifatigue activities. Therefore, there is a critical need to elucidate and predict the qualitative and quantitative properties of this phytochemical compound using the high performance liquid chromatography (HPLC) technique. In this paper, three different nonlinear models including artificial neural network (ANN), adaptive neuro-fuzzy inference system (ANFIS), and support vector machine (SVM),in addition to a classical linear model [multilinear regression analysis (MLR)], were used for the prediction of the retention time (tR) and peak area (PA) for isoquercitrin using HPLC. The simulation uses concentration of the standard, composition of the mobile phases (MP-A and MP-B), and pH as the corresponding input variables. The performance efficiency of the models was evaluated using relative mean square error (RMSE), mean square error (MSE), determination coefficient (DC), and correlation coefficient (CC). The obtained results demonstrated that all four models are capable of predicting the qualitative and quantitative properties of the bioactive compound. A predictive comparison of the models showed that M3 had the highest prediction accuracy among the three models. Further evaluation of the results showed that ANFIS–M3 outperformed the other models and serves as the best model for the prediction of PA. On the other hand, ANN–M3proved its merit and emerged as the best model for tR simulation. The overall predictive accuracy of the best models showed them to be reliable tools for both qualitative and quantitative determination.

## 1. Introduction

Coriander ( *Coriandrum sativum* L., family *Apiceae* ) is an annual plant generally found in Western Asia and Mediterranean Europe [1]. This plant originated in North America and was then distributed and cultivated in various temperate regions of the world. Its leaves are used for flavoring purposes, while the dry seeds are significant due to their various culinary and medicinal applications. The presence of various bioactive compounds gives this planta wide range of pharmacological properties including antihypertensive, neuro-protective, antidepressant, antimutagenic, antiinflammatory, and antioxidant properties as well as anticancer activities[2].

Isoquercitrin is a flavonoid chemical compound that can be extracted from different plant species such as *Mangifera indica* (mango), *Rheum nobile* , *Annona squamosal* , *Camellia sinensis* (tea), and coriander ( *Coriandrum sativum* ) [3]. It has been reported to possesses biological activities including the prevention of thromboembolism as well as anticancer, antiinflammatory, and antifatigue activities [4]. Therefore, it is critically important to determine the bioactive compound by qualitatively identifying its retention time and quantitatively determining its absorbance. High performance liquid chromatography (HPLC) is among the useful techniques deployed todetermine the qualitative as well as quantitative properties of isoquercitrin[5].

There is a need to optimize the parameters necessary to obtain the desired qualitative and quantitative results for this bioactive compound using HPLC [6]. Although there are studies in the literature regarding liquid chromatographic methods for qualitative and quantitative analysis of bioactive compounds, the main drawbacks include the use of large amounts of toxic solvents, long analysis duration, and high analysis cost [7]. For example, Ali et al. [8] investigated the simultaneous determination of irbesartan (IRB) and hydrochlorothiazide (HCTZ) using RP-HPLC in a combined pharmaceutical preparation. They found that the major potential drawback of the study was the long analysis time. Lu et al. [9] reported that benzotriazole and its derivatives, which are persistent contaminants and cosmopolitan chemicals in nature, can be analyzed using a simple, inexpensive, and quick extraction method known as air-assisted liquid–liquid microextraction, coupled with the HPLC technique. This robust hyphenation gives accurate, precise results with good linearity (r > 0.9996). However, the use of a relatively toxic extractant solvent was the major drawback of their study. Xiao-Wang et al. [10] also reported a novel technique that can be used for in vitro models to screen the bioactive ingredients of various traditional Chinese medicines using cell membrane chromatography. The longer analysis time was a disadvantage of their approach. Lian et al. [11] reported an advanced analytical technique for the elucidation of 10 types of prohibited ingredients used in cosmetics. The study involved the preconcentration of the analytes using supramolecular-based dispersive liquid–liquid microextraction prior to analysis using ultra performance liquid chromatography (UPLC) coupled with different mass-spectrometer detectors. Their use of the solidification solvent dodecanal as the extraction solvent, vortexing, and adding another solvent, tetrahydrofuran, as the disperser solvent increased analysis time as well as cost.

Chromatographic methods are complex in nature, which makes it difficult to present a method that can cover all the required parameters necessary to predict the qualitative and quantitative nature of a bioactive compound and to optimize its performance efficiency. Optimization of chromatographic conditions, such as mobile phase composition(concentration of organic modifier), pH, and temperature, is important in order to obtain reasonable and desired qualitative as well as quantitative results. This method involves the optimization of pH, which offers improved selectivity due to the degree of ionization of the solutes, and mobile phase and stationary phase additives, which may affect the pH. It also focuses on optimizing the composition of the mobile phase, which is the ratio of water and organic modifier.

Recently, researchers have been developing green techniques in different fields of chromatography that are capable of modeling and predicting the behavior of chromatographic systems in order to achieve reasonable results within a short time. Although the traditional chromatographic method serves as the fundamental approach, it suffers from certain weaknesses.In contrast, artificial-intelligence–based models have emerged as promising and reliable chemometric approaches in different studies. Veenaas et al. [12] reported that the QSRR method could be used for the prediction of retention time, which can provide an insight into the separation mechanisms. The study stated that prediction of retention time is vital for decreasing the time needed to identify analytes and developing methods, particularly for nontargeted analysis. The results showed that the descriptor selection method and geometry optimization are relatively less effective compared to the chromatographic similarity of the analytes in the training phase. Xiong et al. [13] reported a novel artificial neural network (ANN)-based model, which involves the modeling and prediction of human cancerous tissues using mass-spectrometry imaging (MSI). The reliability of the ANN, MSI, and spatial information provided accurate and precise results. Yuand Cheng [14] reported a method that can be used for quick determination of the authenticity of Chinese-based medicines using near-infrared (NIR) spectroscopy and least squares support vector machines (LSSVM). The results obtained from LSSVM were compared with those of the traditional BP-ANN and SVM, and LSSVM outperformed the other two models in terms of efficiency. Yi-Minget al.[15] reported that partial least squares (PLS) was frequently used for near-infrared spectral quantitative analysis due to its ability to overcome dimensionality as well as the collinearity of spectral data. The application of this model for two NIR datasets showed that the technique provides good prediction performance, and it outperformed the classical models. Amini et al.[16] reported that QSRR was used to determine and predict the retention factor of 160 different pesticides and 25 environmental organic pollutants in grape and wine. Modeling of the relationship between the selected molecular descriptors and retention time was achieved by linear [partial least square(PLS)] and nonlinear [kernel PLS(KPLS) and Levenberg–Marquardt ANN(L-M ANN)] methods. The models were further validated using the cross-validation method. The results showed that L-M ANN outperformed all the other linear and nonlinear models. Similarly, Veenaas et al. [12] reported that the QSRR method could be used for prediction of retention time (tR), which can provide an insight into the separation mechanisms. The study reported that the prediction oftR is vital for decreasing the time needed for analyte identification and method development, especially in nontargeted analysis. The results showed that the descriptor selection method and geometry optimization are relatively less effective than analyzing the chromatographic similarity of the analytes in the training phase. Korany et al. [17] studied the applications of neural networks for HPLC optimization response-surface models, as compared with MLR methods. The results showed that ANN offered significant possibilities for method development in HPLC. Furthermore, the predicted retention capacities of the analytes were better than those obtained using MLR. Ng Boonet al. [18] studied the prediction of gradient elution, as well the application of complex eluent profiles, for both gradient and isocratic eluents for the separation of analytes in ion chromatography (IC). The research focused more on the insightful modeling of tR and peak width for simulating isocratic, gradient, and multistep gradient separations in IC. A more recent study was conducted by D’Archivio [19] using ANN to model the retention factors of 16 different kinds of amino acids using reversed-phase liquid chromatography through the application of different elution modes. The accuracy of ANN prediction was far better than predictions obtained by applying the same data to retention models based on the solution of the fundamental equation of gradient elution [19].

The contribution and novelty of this study is presented in various forms. First, to the best of our knowledge, to date no study has been conducted in the technical literature indicating the chromatographic application of these methods in coriander plant since the inception of AI-based techniques. Secondly, the experimental aim of the current research is to determine the qualitative and quantitative properties of isoquercitrin from coriander plant. These properties were further simulated using data-driven models, and to the best of our knowledge, this is the first study in the literature depicting the application of AI-based models to predict the qualitative and quantitative properties of the phytochemical compound. Subsequently, the study proposes three different nonlinear models (ANN, ANFIS, and SVM) and a classical linear model (MLR) for the prediction of tR and PA for isoquercitrin using HPLC method development.

## 2. Materials and methods

### 2.1. Materials

All chemicals used in the study are HPLC grade and were purchased from Sigma–Aldrich (Sigma-Aldrich Corp., St. Louis, MO, USA).

### 2.2. Instrumentation

An HPLC instrument (Agilent Technologies 1200 series, USA) with a diode-array detector (DAD) was utilized in this experimental study. The bioactive compound was analyzed on an Eclipse XDB-C18 (150mm × 4.6mm, 5µm) reversed-phase column. The mobile phase consisted of a gradient elution system composed of deionized water (D. I) and formic acid (A) and methanol (B). The flow rate was set at 0.9 mL min ^-1^ with a column temperature of 35°C and an injection volume of 10 μL. The analytical wavelength was set at 254 nm for isoquercitrin, and the identification of isoquercitrin was performed by comparing the retention times of the real samples with those of pure standards. Calculations regarding the quantitative result were made using external standardization by measuring peak areas. The detection limit (LOD) and limit of quantification (LOQ) were calculated using standard deviation over slope.

### 2.3. Quantification of isoquercitrin

Standard solutions of isoquercitrin were prepared in methanol at different concentrations: 0, 0.5, 1, 10, 25, 50, 100, 250, 500, 750, and 1000 ppm. The peak areas (absorbance) of the respective concentrations were used to draw the calibration curve.

### 2.4. Extraction of isoquercitrin from coriander leaves

The coriander leaves were collected from Fresh Farm Company in Alayköy, Northern Cyprus. The leaves were then dried, ground, powdered, and weighed. The weighed samples were further extracted using 100 mL methanol and stirred using a magnetic stirrer for 2 h. The obtained extract was then evaporated using a rotary evaporator. The residue was dissolved in water prior to HPLC analysis.

### 2.5. Proposed methodology

**Figure 1:**
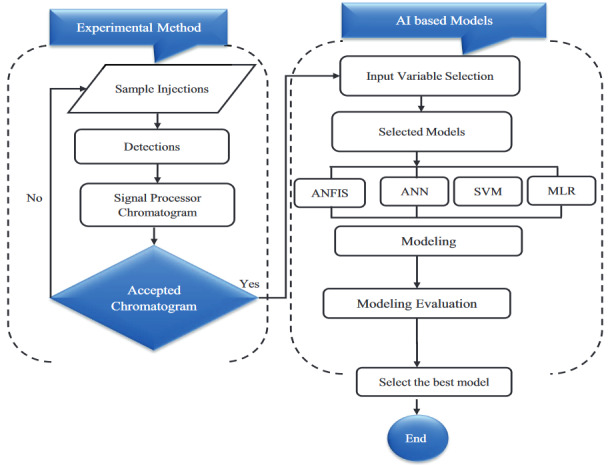
Proposed flowchart of experimental data-driven methods.

For any data-driven method, the knowledge of data science and analysis is crucial; for this reason, the data were collected from our experimental studies. This study proposes the application of four data-driven algorithms including the classical and most commonly used linear model, MLR, and three nonlinear models: ANN (the most widely used data-driven model), ANFIS (a hybrid learning algorithm), and SVM. These models were used to predict the qualitative [i.e.(tR)] and quantitative [i.e. (PA)] properties of isoquercitrin from coriander plant. Concentration of the standards, pH, and composition of mobile phase (methanol and water) were used as the corresponding input variables. The basic motivation for employing different data-intelligence models is the difficulty in understanding whether a specific model is superior to others in practice. Choosing appropriate models for a particular case can be challenging for predictors. This complexity can only be overcome by selecting and comparing different data-driven models, including the linear models, despite their weaknesses in handling highly nonlinear and complex data. Figure 1 presents a flowchart of the methods used for the development of the current study. The input data were collected, preprocessed, and normalized based on Eq.(1) (Figure 1). Normalization of the data was conducted before the model training, and this is usually performed to increase the accuracy and speed of the model. Predictive models such as regression and data-intelligence models are usually evaluated using different numerical indicators, as presented in Section (2.6) below.

(1)y=0.05+(0.95x(x-xminxmax-xmin))

where *y* is the normalized data, *x* is the measured data, and *x*_*max*_ and *x*_*min*_ are the maximum and minimum values of the measured data, respectively.

### 2.6. Artificial neural networks (ANNs)

**Figure 2:**
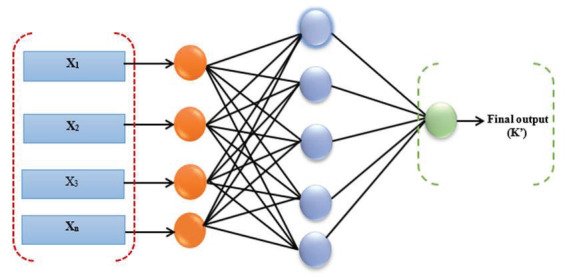
The general architecture of ANN model.

Generally, ANNs are used to build models to aid in understanding the process itself, which is motivated by the configuration of the human brain. TheANN is made of artificial neurons that are considered robust and interconnected processing systems that act together in order to elucidate a certain problem[20]. Artificial neural networks are mostly applied in complex situations that cannot be solved using classical computational methods. The ANN model is a learning algorithm in which the relationships that exist between the predictor and output elements are generated by the data itself. It is important to note that an ANN is proficient enough to learn from wide examples [19]. The efficiency of an ANN is sufficient to resolve an incomplete task and then estimate the outcomes. These two considerable properties of ANNs distinguish it from other data-driven models and lead to its high level of applicability in diverse research areas. The overall architecture of an ANN is shown in Figure 2. The network is composed of various layers of neurons. In order to predict the measurable relationship that exists between input and output variables to a suitable degree of accuracy, it is highly recommended to have at least one hidden layer consisting of a number of nodes [17]. Generally, this model consists of three steps: calibration, validation, and verification.

### 2.7. Adaptive-neuro fuzzy inference system (ANFIS)

The ANFIS is considered a general estimator that can respond to all kinds of complex problems. The ANFIS is a hybrid of adaptive multilayer and feed-forward networks and consists of input–output variables together with fuzzy rule, which is based on the Takagi–Sugeno type. Fuzzier and defuzzifier are the major parts of the fuzzy database system. Fuzzy logic involves the conversion of input data into fuzzy values through the application of membership functions (MFs). Nodes work as MFs and permit modeling of the relations between input and output. There are various types of membership functions, such as triangular, sigmoid, Gaussian, and trapezoidal [21].

Assume the FIS contains two inputs,x and y, and one output,f; a first-order Sugeno fuzzy has the following rules.

(2)Rule 1: ifμ(x)isA1andμ(y)B1thenf1=p1x+q1y+r1

(3)Rule 1: ifμ(x)isA2andμ(y)B2thenf2=p2x+q2y+r2

*A*_1_ , *B*_1_ , *A*_2_ , *B*_2_ , parameters are membership functions for x and y, and inputs *p*_1_ , *q*_1_ , *r*_1_ , *p*_2_ , *q*_2_ , *r*_2_ are outlet function parameters. The structure and formulation of ANFIS follows a five-layer neural network arrangement. Refer to Lu et al. [9] for more information about ANFIS.

### 2.8. Multilinear regression (MLR)

Generally, regression models predict the extent of correlation between input and output parameters as well as the relationship that exists between them. Linear regressions are generally fitted using a least squares approach, although other methods could be employed, such as minimizing the lack of fit in some of the norms or reducing the penalized version loss of the least square function, as in ridge regression. Primarily, linear regression is categorized into two major divisions: multiple and simple linear regression. A linear regression is considered simple if it is aimed at predicting the correlation between a single output by using a single input variable. However, if the aim is to estimate the correlation between two or more input variables in order to determine a single criterion variable, this model is referred to as MLR. In MLR each value of the input parameter is associated with a value of the output variable. Multilinear regression is the most widely utilized form of linear regression and has been used in various areas of study. It is worth mentioning that MLR shows a correlation in terms of a straight line which can best estimate all the data points involving both output and target variables [20]. The general form of the MLR model is shown in Eq. (4).

(4)y=b0+b1x1+b2x2+…bixi

where *x*_*1*_ ,is the value of the *i* th predictor, *b*_0_ is the regression constant, and *b*_*i*_ is the coefficient of the *i* th predictor.

### 2.9. Support vector machine

In 1995 Vapnik proposed the idea of learning in the context of the SVM, which provides the desired mechanism for solving problems that involve classification, prediction, pattern recognition, and regression. The SVM works according to the concept of machine learning and is a data-driven model[21]. The two major functions of SVM are statistical learning theory and structural risk minimization. This helps SVM offer insight that differs from ANN, as itreduces error, redundancy of data, and complexity and increases the general performance of the system. Support vector machine can be classified into linear support vector regression and nonlinear support vector regression [22]. This means that support vector regression (SVR) is a form of SVM based on two basic structural layers: the first layer is a kernel function weighting on the input variable while the second function is a weighted sum of kernel outputs [23]. In SVM the linear regression is first fitted on the data, and then the outputs go through a nonlinear kernel to identify the nonlinear pattern of the data. The calibration data is: {( *x*_*i*_ *, d*_*i*_ )} *N*_*i*_ ( *x*_*i*_ is the input vector, *d*_*i*_ is the actual value, and *N* is sum of the data), where overall SVM function is given as:

(5)y=f(x)=ωΦ(xi+b

where *φ(x*_*i*_ *)* indicates feature spaces, nonlinearly mapped from input vector *x* .

(6)Minimize12∥ω2+C(iN∑(ξi+i*ξ))

**Figure 3:**
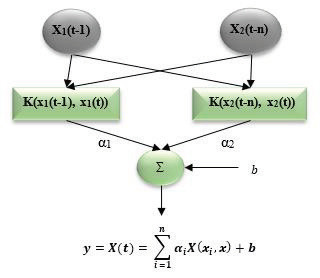
The Architecture of SVM Algorithms.

Subject to:{ωiΦ(xi)+bi-di≤ε+i*ξdi-ωiΦ(xi)+bi≤ε+i*ξξi,i*ξ,i=1,2,...,N

where 1/2 ∥ ω ∥ ^2^ the weights vector norm, and C is referred to the regularized constant. The general conceptual model structure of SVM is illustrated in Figure 3. The parameters of the Lagrange multipliers are defined as *α*_*i*_ and *α*_i_^*^ . Vector *w* in Eq. (7) can be calculated after finding the problem solution of optimization.

(7)ω*=i=1N∑(αi-i*α)Φ(xi)

Therefore, the overall form of SVM informs Eq.(8).

(8)f(x,iα,i*α)=i=1N∑(αi-i*α)K(x,xi)+b

where *k (x*_*i*_ *, x*_*j*_ *)* is the kernel function, and *b* is the bias term. The radial basis function (Gaussian) is the most common kernel function and is expressed as:

(9)k(x1,x2)=exp(-γ∥x1-x2∥2)

where γ is the kernel parameter.

### 2.10. Evaluation criteria for data-driven models

Generally, for any form of data-driven approach, performance accuracy is evaluated using various criteria based on a comparison between the predicted and measured values. In this study, the determination coefficient (DC) as a goodness-of-fit, correlation coefficient (CC), and two statistical errors, root mean-squared error (RMSE) and mean-squared error (MSE), were used for the evaluation of the models.

(10)DC=1-j=1N∑[(Y)obs,j-(Y)com,j]2j=1N∑[(Y)obs,j-(Y)-com,j]2

(11)CC=i=1N∑(Yobs-Y-obs)(Ycom-Y-com)i=1N∑(Yobs-Y-obs)2i=1N∑(Ycom-Y-com)2

(12)RMSE=i=1N∑(Yobsi-Y-comi)2N

(13)MSE=1Ni=1N∑(Yobsi-Ycomi)2

where N, *Y*_*obsi*_ , *Y* , and *Y*_*comi*_ *a* are data number, observed data, average value of the observed data, and computed values, respectively.

### 2. 11. Data set description and validation of the models

In a data-driven method, the primary objective is to fit the models to a given data set based on the employed indicators in order to produce a reliable prediction of the unknown data set. Considering issues such as overfitting, satisfactory training performance is not always in agreement with the testing performance. In the validation process, different types of validation approaches can be applied including cross-validation, which is called k-fold cross-validation; others are [24] holdout, leave one out, and so on. The major advantage of the k-fold cross-validation mechanism is that in every single round, the validation set and the training sets are independent [25]. As stated above, the data is further divided into categories; 75% for the calibration (training) and 25% for the testing (verification) stage. Considering the k-fold cross-validation, it is important to note that other validation methods can be applied to the data set [26]. Furthermore, the data in the current study was collected over a period of two months, and the data set was composed of 64 instances for each variable.

## 3. Results and discussion

### 3.1. Experimental results

**Figure 4:**
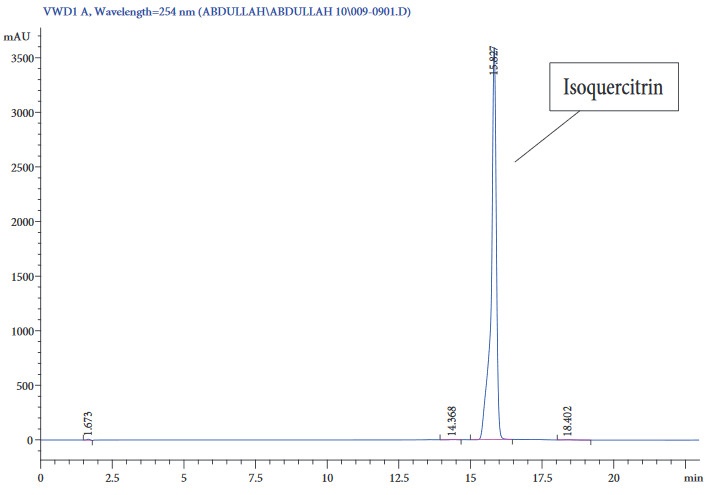
Chromatogram of Isoquercitrin standard.

**Figure 5:**
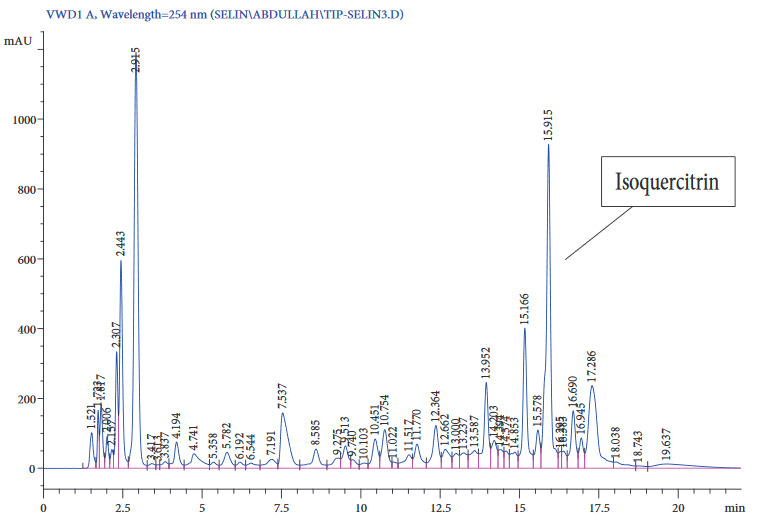
Chromatogram of Coriandrumsativum extract.

The chromatogram obtained from the HPLC analysis above showed a well-resolved peak with little or no interference for the standard as well as the real sample. The chromatogram of the standard and the sample extract are shown in Figures 4 and 5, respectively. Isoquercitrin was found to have an average retention time of 15. The absorbance of the leaf extract analyte is shown in the Figures 4 and 5 for the standard and sample extracts, respectively.

It is important to note that all samples analyzed possessed greater amounts of isoquercitrin than other bioactive ingredients of the coriander plant. According to Yunjeong et al., coriander is not the only source of isoquercitrin. There are other natural sources, particularly in plants, that contain large amounts of isoquercitrin. In the abovementioned studies, 11 different plant families together with other natural sources were examined, and interestingly, most of them contained the analyte in high quantities. The major sources were onions, tea, and tartary buckwheat bran [27]. The isoquercitrin content revealed by their research is similar to the findings of this study.

### 3.2. Artificial intelligence results

Data-driven approaches (ANN, ANFIS, SVM, and MLR) were analyzed in order to predict the qualitative and quantitative properties of isoquercitrin using the HPLC technique. Before model calibration, data were analyzed statistically, as shown in Table 1. Statistical analysis is generally used to understand the science of the data in order to navigate common problems that can lead to incorrect results and to facilitate decision-making based on raw data.

**Table 1:** Statistical analysis.

Mean	251.8593	74.0625	25.9375	3.515625	16328.94	15.35734
Standard Deviation	342.4210	13.4066	13.4066	0.560593	21821.09	1.104323
Minimum	0.0000	55.0000	10.0000	2.8000	0.0000	11.9100
Maximum	1000.0000	90.0000	45.0000	4.3500	67281	17.3300

The Spearman–Pearson correlation shows the relationship between variables and their fitness using a linear function. The correlation strength is independent of the sign or direction. A positive coefficient shows that, as the first variable increases, the second variable also increases; a negative correlation shows an inverse relationship between the parameters, in other words, when the first parameter increases, the second parameter decreases, and vice versa [28]. As seen in Table 2, there is a strong correlation between the concentration of the standard and the peak area (PA) (R=0.998501), while a lesser correlation was observed between concentration and tR. Table 2 also shows a positive correlation between tR and MP-B, pH, and PA, while there is a negative correlation between tR and concentration and MP-A.

**Table 2:** Spearman Pearson correlation analysis.

Parameters	Concentration	MP- A	MP-B	pH	PA	tR
Concentration	1					
MP-A	–0.39241	1				
MP-B	0.392407	–1	1			
pH	–0.09277	0.046006	–0.04601	1		
PA	0.998501	–0.38588	0.385881	–0.09454	1	
tR	–0.10739	–0.09525	0.095247	–0.08569	–0.08216	1

For development of the data-driven models, MATLAB 9.3 (R2017a) was used in the ANN, SVM, and ANFIS models, while the deterministic linear MLR model was developed using the simulation tool in EViews 9.5 software. According Usman et al. [25], the proper number of nodes in the hidden layer for identification of the optimum number of hidden layers ranges from (2n1/2 + m) to (2n+1), where n is the number of input neurons, and m is the number of output neurons. Hence, 3–21 was the range of hidden neurons in the ANN model for predicting the qualitative and quantitative properties of the phytochemical compound by HPLC method. According to Abba et al., obtaining suitable hidden nodes is the crucial aspect in any ANN modeling, as it helps to prevent overfitting caused by different factors. As reported in several works in the field of science and engineering, there is no specific standard method for determining the appropriate number of hidden neurons [29]. Furthermore, appropriate and optimal determination of parameters (Cε, γ) in the SVM models is very important in choosing the best structure for the models. In this research, optimal values were obtained by employing the kernel function of the grid procedure, as suggested by Pham et al. [30]. For ANFIS modeling, various types of MFs and epoch iterations were explored using trial and error to identify the best structure. Table 3 shows the results of the performance analysis for the four data-driven models.

**Table 3:** Results of the ANN, ANFIS, SVM and MLR models.

		Calibration				Verification			
Variables	Models	DC	RMSE	MSE	CC	DC	RMSE	MSE	CC
PA	ANN-M1	0.9966	0.0183	0.0003	0.9983	0.9984	0.0054	0.0003	0.9992
	ANN-M2	0.9413	0.0761	0.0058	0.9702	0.9758	0.0211	0.0044	0.9878
	ANN-M3	0.9978	0.0219	0.0005	0.9989	0.9991	0.0041	0.0002	0.9996
tR	ANN-M1	0.8570	0.2295	0.0527	0.9257	0.8939	0.0754	0.0568	0.9454
	ANN-M2	0.8639	0.2346	0.0550	0.9295	0.8476	0.0903	0.0816	0.9207
	ANN-M3	0.9988	0.0045	0.0022	0.9994	0.9987	0.0063	0.0036	0.9993
PA	ANFIS-M1	0.9993	0.0126	0.0002	0.9996	0.9994	0.0033	0.0001	0.9997
	ANFIS-M2	0.9995	0.0103	0.0001	0.9997	0.9990	0.0006	0.0000	0.9995
	ANFIS-M3	0.9990	0.0002	0.0001	0.9995	0.9998	0.0002	0.0001	0.9999
tR	ANFIS-M1	0.9821	0.1626	0.0264	0.9910	0.9227	0.0643	0.0414	0.9606
	ANFIS-M2	0.9669	0.1055	0.0111	0.9833	0.9516	0.0509	0.0259	0.9755
	ANFIS-M3	0.9990	0.0005	0.0039	0.9995	0.9980	0.0004	0.0084	0.9990
PA	SVM-M1	0.9976	0.0228	0.0005	0.9988	0.9972	0.0072	0.0005	0.9986
	SVM-M2	0.9986	0.0171	0.0003	0.9993	0.9982	0.0057	0.0003	0.9991
	SVM-M3	0.9986	0.0171	0.0003	0.9993	0.9983	0.0057	0.0003	0.9991
tR	SVM-M1	0.9110	0.1930	0.0373	0.9545	0.9155	0.0673	0.0452	0.9568
	SVM-M2	0.9508	0.1908	0.0364	0.9751	0.9173	0.0665	0.0443	0.9578
	SVM-M3	0.9671	0.1927	0.0371	0.9834	0.9225	0.0644	0.0415	0.9605
PA	MLR-M1	0.9987	0.0168	0.0003	0.9993	0.9981	0.0059	0.0004	0.9990
	MLR-M2	0.9986	0.0171	0.0003	0.9993	0.9982	0.0057	0.0003	0.9991
	MLR-M3	0.9986	0.0171	0.0003	0.9993	0.9983	0.0056	0.0003	0.9991
tR	MLR-M1	0.9211	0.1933	0.0373	0.9598	0.9160	0.0670	0.0450	0.9571
	MLR-M2	0.9308	0.1908	0.0364	0.9648	0.9173	0.0665	0.0443	0.9578
	MLR-M3	0.9411	0.1931	0.0373	0.9701	0.9232	0.0641	0.0411	0.9608

**Figure 6:**
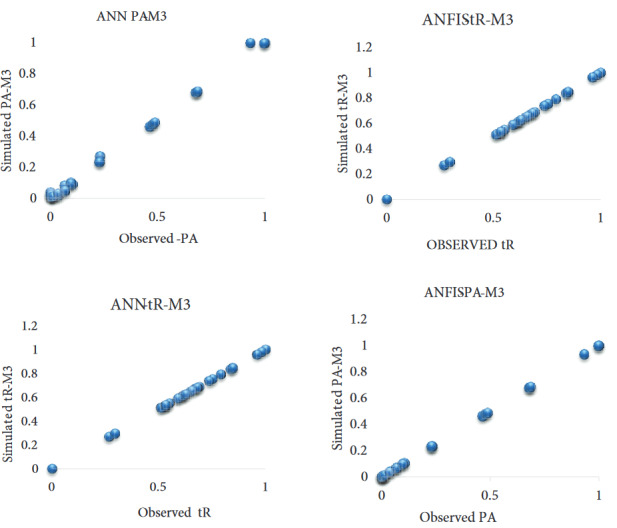
Scatter plots for ANN, and ANFIS, (a) Peak area (PA) and (b) Retention time (tR).

It is clear that the nonlinear models (ANN, SVM, and ANFIS) outperformed the traditional linear regression model (MLR) in the predictive comparison among the four models. Table 3 further demonstrates that all four models are capable of predicting the qualitative (tR) and quantitative (PA) properties of the bioactive compound. It is important to note that the performance efficiency of all four models, in terms of DC, CC, MSE, and RMSE, shows a satisfactory and reliable accuracy. This may be due to the cross-validation process conducted before model calibration, which is a very significant component of model evaluation [31]. Table 3 also shows that M3, with four input parameters, provided the highest prediction accuracy among the four data-driven methods in terms of DC, CC, MSE, and RMSE, for the prediction of PA and tR. Among these models,ANFIS-M3 with DC (0.9998), RMSE (0.0002), MSE (0.0001), and CC (0.9999) values in the verification phase had the highest levels of accuracy when compared to ANN-M3, SVM-M3, and MLR-M3 for the prediction of PA.However, for the prediction of tR in the verification phase,ANN-M3 outperformed the other three models(ANFIS-M3, SVM-M3, and MLR-M3) in DC (0.9987), RMSE (0.0063), MSE (0.0036), and CC (0.9993) values, and this result is in line with previous findings [31–33]. The predictive precision relating to DC showed that ANN outperformed the other three models (ANFIS, SVM, and MLR) and increased the prediction accuracy up to 0.07%, 8%, and 7%, respectively. The predicted results were displayed graphically using a scatter plot in order to demonstrate the goodness-of-fit between the experimental and predicted values for the two best models (ANN and ANFIS) (Figure 6). It is apparent from the scatter plots that both ANN and ANFIS models demonstrated good fitting agreement between the experimental and predicted values. The higher prediction skill of PA can be attributed to the high correlation values between variables (Table 2).

**Figure 7:**
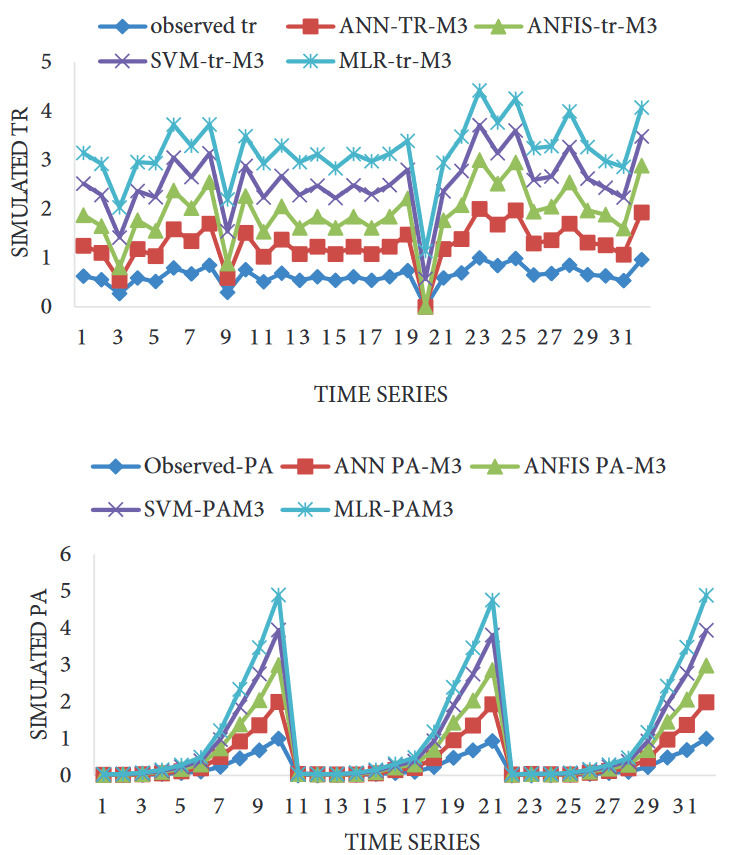
Time series for the prediction of (a) PA and (b) tR.

Figure 7 shows the response time series plot for PA and tR. According to the plot, the extent of spread values between the experimental and predicted values proved the Table 3 data above.

## 4. Conclusion

This work examined the qualitative and quantitative properties of isoquercitrin from coriander plant using the HPLC method to simulate tR and PA, respectively. The analyte was determined from a leaf extract, and a considerable amount was found. The work further explored the application of data-driven methods including a classical linear model (MLR) and three AI-based models (ANFIS, ANN, and SVM),in order to simulate tR and PA of isoquercitrin from the coriander plant using HPLC technique. The concentration of the standard, composition of the mobile phases, and pH were used as input variables. The results obtained indicated that all four data-driven approaches are capable of simulating the tR and PA of the analyte. Furthermore, comparison of the models demonstrated that M3 offered the highest level of predictive accuracy, as shown in Table 3. Further comparative analysis of the results showed that ANFIS-M3 outperformed the other models for the prediction of PA. However, ANN outperformed the other three models (ANFIS, SVM, and MLR) and increased accuracy up to 0.07 %, 8 %, and 7 %, respectively, for the prediction of qualitative properties of the analyte (tR).
